# Donor Specific Anti-HLA Antibody and Risk of Graft Failure in Haploidentical Stem Cell Transplantation

**DOI:** 10.1155/2016/4025073

**Published:** 2016-01-24

**Authors:** Piyanuch Kongtim, Kai Cao, Stefan O. Ciurea

**Affiliations:** ^1^Department of Stem Cell Transplant and Cellular Therapy, The University of Texas MD Anderson Cancer Center, Houston, TX 77030, USA; ^2^Division of Hematology, Department of Internal Medicine, Faculty of Medicine, Thammasat University, Pathumthani 12120, Thailand; ^3^Department of Laboratory Medicine, The University of Texas MD Anderson Cancer Center, Houston, TX 77030, USA

## Abstract

Outcomes of allogeneic hematopoietic stem cell transplantation (AHSCT) using HLA-half matched related donors (haploidentical) have recently improved due to better control of alloreactive reactions in both graft-versus-host and host-versus-graft directions. The recognition of the role of humoral rejection in the development of primary graft failure in this setting has broadened our understanding about causes of engraftment failure in these patients, helped us better select donors for patients in need of AHSCT, and developed rational therapeutic measures for HLA sensitized patients to prevent this unfortunate event, which is usually associated with a very high mortality rate. With these recent advances the rate of graft failure in haploidentical transplantation has decreased to less than 5%.

## 1. Introduction

Allogeneic hematopoietic stem cell transplantation (AHSCT) using one human leukocyte antigen (HLA) haplotype matched first-degree relative donor (haploidentical donor) represents an alternative treatment for patients with hematologic malignancies who lack HLA-matched related or unrelated donor. Historically, the main limitations of this treatment modality were high rate of graft failure (GF) and graft-versus-host disease (GVHD), which occur due to intense alloreactive reactions related to the major HLA mismatch between the recipient and the donor. Although several approaches have been developed which aimed to partially deplete T cells in the graft and decrease graft-versus-host alloreactivity, GF remains a major obstacle [1–3]. While increased rate of engraftment has occurred with the use of “megadoses” of hematopoietic stem cells (over 10 million CD34^+^ cells/kg with a very low T cell content) (1 × 10^4^ CD3^+^ cells/kg) [4, 5], approximately 10–20% of patients still developed GF [6–8]. The increased risk of GF following haploidentical stem cell transplant (haploSCT) is due, in part, to an enhanced susceptibility of the graft to regimen-resistant host natural killer (NK) cell- and T lymphocyte-mediated rejection against mismatched donor cells [9, 10]. In addition to T cell- and NK-cell-mediated graft rejection (cellular rejection), antibody-mediated rejection (humoral rejection) occurring either by antibody-dependent cell-mediated cytotoxicity or complement mediated cytotoxicity has been described [11, 12]. Preformed donor-specific anti-HLA antibodies (DSAs) present at the time of transplant have been shown to be correlated with graft rejection and decrease survival in solid organ transplantation [13–16]. Therefore, lymphocyte crossmatch tests have been developed for prediction of graft rejection [17, 18] and became mandatory in solid organ transplant according to the American Society for Histocompatibility and Immunogenetics (ASHI). In AHSCT setting, there has been reported that a positive crossmatch for anti-donor lymphocytotoxic antibody associated strongly with GF, mainly in mismatched or haploSCT patients [19, 20]. Although a lymphocyte crossmatch is an effective tool to evaluate alloimmunization and potential donor-recipient incompatibility, the procedure is labor intensive and may detect non-HLA antibodies, which may not be associated with transplant outcome since there is no data to confirm the importance of these antibodies to date. Over the recent years, several methods have been developed to more precisely detect and characterize DSAs in AHSCT recipients [21, 22], and also the clear association between the presence of these antibodies and GF has been confirmed especially in mismatched and haploSCT patients [14, 23, 24]. Still, the mechanisms by which DSA may cause GF in AHSCT remain an area of active research.

Here we review the potential mechanisms and clinical importance of DSAs on GF in haploSCT, as well as treatment modalities used for DSA desensitization before transplant to abrogate the risk of GF and improve transplant outcomes.

## 2. Mechanisms of Graft Rejection in Haploidentical Stem Cell Transplantation

Engraftment failure rate has been approximately 4% in AHSCT using matched unrelated donors and about 20% in umbilical cord blood (UCB) or T cell-depleted haploSCT [25, 26]. The common cause of GF is host immunologic reaction against donor cells, so called graft rejection. Graft rejection following haploSCT is generally attributed to cytolytic host-versus-graft reaction mediated by host T and/or NK-cells that survived the conditioning regimen. However, antibody-mediated graft rejection (otherwise known as humoral rejection) has been increasingly recognized in the past decade.

### 2.1. Cellular-Mediated Graft Rejection

The resistance to engraftment of AHSCT was thought to be mediated primarily by recipient T lymphocytes which depends on the genetic disparity between the donor and recipient and the status of host antidonor reactivity [27]. This makes mismatched and haploSCT recipients likely more susceptible to develop graft rejection compared with matched AHSCT due to stronger alloreactive reactions in this setting. It has been found in animal model of stem cell transplantation that antidonor cytotoxic T cells sensitized to major and minor histocompatibility (MHC) antigens confer resistance against allogeneic bone marrow stem cells [28]. This finding also has been confirmed in clinical studies of AHSCT in patients with severe aplastic anemia, in which the presence of radioresistant antidonor cytotoxic T cell populations sensitized to donor MHC antigens through repeated blood transfusions is associated with a higher incidence of graft rejection and death [29]. Nevertheless, the molecular bases underlying T cell-mediated graft rejection remain incompletely defined.

NK-mediated graft rejection also has been demonstrated in animal models [9, 30, 31]. In preclinical models of bone marrow transplantation, radioresistant host NK-cells are also capable of lysing donor hematopoietic cell targets and rejecting bone marrow grafts, especially those that lack expression of MHC class I antigens [32]. Evidence that NK-cells mediate resistance to engraftment in clinical AHSCT is lacking, due in part to the difficulty of discriminating T cell- from NK-cell-mediated resistance in humans.

In haploSCT, the use of myeloablative conditioning chemotherapy and high-dose posttransplant cyclophosphamide can diminish these cellular-mediated immune reactions due to the fact that both human T cells and NK-cells are highly sensitive to cyclophosphamide, which is now commonly used after haploSCT to prevent GVHD [33].

### 2.2. Antibody-Mediated Graft Rejection

Antibody-mediated graft rejection has been a major obstacle and well recognized cause of rejection and organ dysfunction in solid organ transplants, especially in kidney transplantation, because transplanted kidneys are highly susceptible to antibody-mediated injury [34–36]. In animal models of AHSCT, preformed antibodies present at the time of marrow infusion in multitransfused mice, rather than primed T cells, have been shown to be a major barrier against marrow engraftment resulting in rapid graft rejection within a few hours in allosensitized recipients of MHC mismatched bone marrow transplantation while T cell-mediated graft rejection takes much longer [12, 37]. The risk of antibody-associated graft rejection in human depends on antigen density on the target and capacities of the antibody Fc-domain. While many types of preformed antibodies can be detected in alloimmunized stem cell transplant recipients, only antibodies against donor HLA antigens have been shown to have clinical significance [38–40].

## 3. Role of Complement System in DSA-Mediated Graft Rejection

Antibody-mediated BM failure after AHSCT can occur either by antibody-dependent cell-mediated cytotoxicity or by complement mediated cytotoxicity [41]. Evidence from studies in cardiac and renal transplant patients has shown that complement system is activated in the transplanted organ during rejection and can be detected by measuring the products of complement activation in the patients' blood and urine as well as in the transplanted organ itself [42–45]. In haploSCT setting, we recently found that DSAs that bind complement, detected by the C1q assay, the first component of the classical complement pathway, plays an important role in the development of graft rejection in haploSCT recipients. In this study, the presence of C1q-fixing DSA was found in 9 of 22 patients who had DSAs and was associated with a significantly higher rate of GF compared with patients who had DSAs but negative C1q. Moreover, 4 patients who became negative C1q after treatment with plasmapheresis and immunosuppressive therapies before transplant could engraft with donor cells successfully while 5 patients who remained positive C1q experienced GF [46]. Previous studies by Chen showed that there is no predictability by IgG mean fluorescence intensity (MFI) as to which of the antibodies will bind C1q because fixation is independent of MFI values [47]. However, most patients who had positive C1q in our study had higher median MFI of DSAs (all more than 5,000 MFI) compared with those who had negative C1q [46]. These results suggest that the possibility of complement fixation might depend on both ability and level of DSAs.

## 4. Prevalence and Risk Factors for the Development of DSAs in Haploidentical Stem Cell Transplantation

Anti-HLA antibodies can be found in healthy individuals as a consequence of allosensitization during pregnancy or related to either previous transplant with mismatched donor or multiple transfusions of blood products and the clinical significance of anti-HLA antibodies is well known in the field of transfusion medicine. The presence of anti-HLA antibodies in patients is one of the major causes of platelet refractoriness [57]. On the other hand, anti-HLA antibodies present in blood products have been shown to be a major cause of transfusion-related acute lung injury (TRALI) [58]. According to previous reports in healthy blood donors, anti-HLA antibodies could be identified up to 50% depending on sensitivity of the test used for screening [59–61]. The reported prevalence of anti-HLA antibodies is of approximately 20–25% in patients undergoing haploSCT [40, 48, 49].

Despite a high prevalence of anti-HLA antibodies reported in AHSCT patients, these anti-HLA antibodies might not be specific to donor HLA antigens. A delay in recognizing this as a major cause of GF in AHSCT could be because hematopoietic transplantation has been performed mostly with a high degree of HLA matching between the donor and recipient. The increasing use of mismatched donors (haploidentical, cord blood, and mismatched unrelated donors), in addition to improvements in detection techniques, has facilitated recognizing DSAs as a major cause of graft rejection in stem cell transplantation. With the use of highly sensitive solid-phase immunoassays, DSAs were identified in up to 24% of stem cell transplant recipients [3, 23, 24, 39, 48, 51, 62]. While, overall, in haploSCT the prevalence of DSAs may range between approximately 10 and 21% [22, 46, 48, 49], this proportion is highly dependent on the recipient's gender with very low prevalence in male recipients (5%) as compared with female recipients (86%) [43]. Anti-HLA antibodies detected in female patients are much more often DSAs in the settings of “child-to-mother” haploSCT compared to the settings of CBT [22, 50]. It is because those anti-HLA antibodies are the results of sensitization during pregnancies by offspring's HLA itself and it makes it often difficult to locate a donor who is not a target of anti-HLA antibodies. Thus it is particularly important to establish an effective desensitization protocol in the setting of haploSCT.

A few studies evaluated transplant outcomes in relation to non-donor-specific anti-HLA antibodies (non-DSAs) in various donor types of AHSCT [38–40]. Takanashi and colleagues reported a similar rate of engraftment in cord blood stem cell transplant recipients who had anti-HLA antibodies without the corresponding HLA in the transplanted cord blood compared with recipients without anti-HLA antibodies, while rate of engraftment in recipients who had anti-HLA antibodies corresponding with donor cord blood HLA (DSAs) was significantly lower [38]. Similar results were found in the study by the Eurocord group, which reported no difference in neutrophil engraftment after single or double UCB transplants in 32 recipients with non-DSAs, compared to 158 patients without HLA antibodies [39]. Also, in a retrospective study of recipients of matched unrelated stem cell transplants, we found that alloimmunization as such did not cause a significant increase risk of GF unless antibodies were directed against the donor HLA antigens, suggesting that DSA is the key to the development of GF in AHSCT [40].

It is well recognized in solid organ transplantation that repeated transfusion is a major risk factor of developing DSAs [63, 64]. DSA developed after transfusion is also an important barrier of successful engraftment in patients with severe aplastic anemia [11] and other thalassemia or hemoglobinopathies [65].

Additionally, there is a strong evidence to suggest that female sex and pregnancy confer a significant risk for allosensitization, and this risk is further increased with a higher number of pregnancies. Our group has formerly observed a striking association between the sex of patients who experience GF and the development of allosensitization. In our study of haploSCT, we found that all patients who developed DSAs were multiparous young women with a median of 3 pregnancies; 30% of women versus 12% of men had DSAs (*P* < 0.0001) and 7 of 8 patients with DSAs were women, all of whom except 1 had at least 2 prior pregnancies. When the presence of DSAs was evaluated in women with no pregnancies compared with the male recipients, no significant association was identified. Although the majority of allosensitized individuals in this study were women, 12% of patients with anti-HLA antibodies were men, suggesting that other factors are associated with the development of anti-HLA antibodies in these patients, most likely related to transfusion of blood products [22].

## 5. Testing for Anti-HLA Antibodies

### 5.1. DSA Testing

Pretransplant sera of patient are tested for anti-HLA class I and class II antibodies using multianalyte bead assays performed on the Luminex platform including LABScreen® PRA, LABScreen Mixed methods for screening; the binding level of DSA is determined by the LABScreen Single Antigen bead assay (One Lambda, Part of Thermo Fisher Scientific, Canoga Park, California, USA) per manufacturer's instructions and results are expressed as mean fluorescence intensity (MFI). Briefly, 5 *μ*L of mixed beads, HLA class I and class II single antigen beads, is added to 20 *μ*L of sample serum and incubated for 30 min at room temperature (RT) in the dark with gentle shaking. After washing with wash buffer three times, 100 *μ*L of goat anti-human IgG secondary antibody conjugated with R-phycoerythrin (PE) is added and the samples are incubated in the dark for 30 min at RT. After washing three times, the samples are read on Luminex-based LABScan*™* 100 flow analyzer. Antibody specificity and binding level are analyzed and determined through HLA Visual or HLA Fusion software from the manufacturer.

### 5.2. C1q Testing

Complement binding antibodies are detected for patients with DSA using the C1q assay. The complement component (C1q) bound by the antigen-antibody complex is detected with R-PE labeled anti-C1q antibody. Fluorescence intensity is measured using Luminex-based LABScan 100 flow analyzer. DSA specificity and binding level are determined by the C1qScreen*™* assay per manufacturer's instructions [One Lambda, Part of Thermo Fisher Scientific (Canoga Park, California, USA)]. Briefly, 5 *μ*L of human C1q and 5 *μ*L of HLA class I and class II single antigen beads are added to 5 *μ*L of heat-inactivated sample serum and incubated for 20 min in dark at RT, followed by adding 5 *μ*L of R-PE labeled anti-C1q antibody and incubation for 20 min in dark at RT. The samples are read and C1q specific antibody specificity and binding levels are analyzed and determined.

## 6. DSA and Haploidentical Stem Cell Transplant Outcomes

Multiple investigators have demonstrated that DSAs are associated with primary GF in either mismatched related (haploidentical), matched, and mismatched unrelated donor or UCB transplants (Table 1). This association appears more discernable in haploSCT presumably due to the close relationship and higher likelihood of sharing the mismatched HLA antigens with DSAs against the immediate family.

Back in 2009, our group initially showed that DSAs are associated with primary GF in AHSCT with mismatched donors [22]. We tested 24 consecutive patients including a total of 28 haploSCTs with “megadoses” of CD34^+^ stem cells for the presence of DSAs determined by a highly sensitive and specific solid-phase/single antigen assay. DSAs were detected in 5 patients (21%). Three out of 4 (75%) patients with DSAs prior to transplant failed to engraft, compared with only 1 out of 20 (5%) without DSAs (*P* = 0.008). All 4 patients who experienced primary GF had second haploSCT and 1 patient who had persistent high titer of DSAs developed a second GF, while 2 out of 3 engrafted patients had the absence of DSAs [22]. Patients in this study had DSAs directed against high-expression HLA loci, including class I HLA antigens (HLA-A and HLA-B) and class II (HLA-DRB1) antigens. In a later study, we found that anti-HLA antibodies directed against low-expression loci (HLA-DPB1 and HLA-DQB1) are also associated with graft rejection, however, to a lower extent. In our large prospectively tested patients for HLA antibodies of 592 matched unrelated AHSCT recipients, anti-HLA antibodies that were not reactive with donor loci were identified in 116 patients (19.6%), whereas DSAs were found only in 8 patients (1.4%) in this population, all directed against the HLA-DPB1 molecule. Overall, GF occurred in 19 of 592 patients (3.4%), including 16 of 584 (2.7%) patients without DSAs compared with 3 of 8 (37.5%) patients with DSAs (*P* = 0.0014). As noted above, we have found that the presence of anti-HLA antibodies in the absence of DSAs did not predict graft failure. In multivariate analysis, DSA was the only factor that predicted GF in these patients [40]. Recently we reported outcomes of 122 patients receiving haploSCT including 22 patients with DSAs. Results from this study were consistent with the previous reports, a significantly higher proportion of DSA-positive patients experienced GF (32%) compared with DSA negative patients (4%; *P* < 0.001) [46].

In another study in haploSCT by Yoshihara and colleagues, the authors tested anti-HLA antibodies in 79 patients receiving haploSCT. Among 79 screened patients, 16 (20.2%) were anti-HLA antibodies-positive, including 5 non-DSA-positive and 11 DSA-positive patients. The cumulative incidence of donor neutrophil engraftment was significantly lower in DSA-positive patients than in DSA-negative patients (61.9 versus 94.4%, *P* = 0.026) [48]. Furthermore the most recent study by Chang and colleagues also confirmed a significantly higher rate of primary graft rejection (20% versus 0.3%) and poor graft function (27.3% versus 1.9%) in haploSCT who developed DSAs before transplant compared with recipients without DSAs.

The clinical importance of DSAs has also been confirmed in other donor types of AHSCT. In a retrospective case controlled study by Spellman and colleagues, they have demonstrated that the prevalence of DSAs was higher in a group of mismatched unrelated donor-recipients who suffered graft rejection than in a control group that engrafted. Among the 37 recipients who failed to engraft, 9 (24%) had DSAs against HLA-A, HLA-B, or HLA-DP, whereas DSA was identified in only 1 of 78 patients in the control group who successfully engrafted [23].

Same results have also been demonstrated in some studies in patients receiving umbilical cord stem cell transplant as summarized in Table 1.

Besides GF, some investigators have shown that patients with DSAs had significantly lower event-free survival as well as overall survival compared with those without DSAs [24, 39, 50]. Though the results from these studies have clearly confirmed that the presence of DSAs influences graft outcomes and survival in haploSCT, we need to bear in mind that different cut-off levels of DSAs as well as different methods of DSAs detection were used in these studies. The definition of a threshold for DSAs, according to MFI, is a premise for analyzing the association of DSAs with GF. In a case-control study conducted by us, MFI of 500 or more was considered positive [40], while, in haploSCT, MFI values of more than 1500 or 5000 were defined as significant by our group [22] and by Yoshihara et al. [48], respectively. An important difference between these two studies is that our study was done in patients treated with a T cell-depleted graft, while the second one was done in patients treated with a T cell replete graft with ATG or intensified GVHD prophylaxis. It is possible that stem cells without T cells are more exposed to the HLA antigens as the only targets available for the DSAs and by the lack of contribution of donor T cells to engraftment and eradication of recipient's alloreactive T cells. Recently, Chang and colleagues also showed that positive DSA at MFI of 10,000 or more was correlated to primary graft rejection while MFI of 2,000 or more was strongly associated with primary poor graft function [49]. So far the conclusion from these published studies is that a very strong titer of DSA, which may be revealed by serum dilution or titration for those false-low or false negative antibodies defined by the MFI in the solid-phase immunoassays, poses an absolute contraindication to transplantation (in the absence of treatment), whereas very weak antibodies may be considered as a relative contraindication for transplantation. Although the standard cut-off level of DSAs that is considered safe for transplant still needs to be determined, it is likely that other transplant factors need to be taken into consideration.

## 7. Desensitization Therapy for Allosensitized Recipients

**Figure 1:**
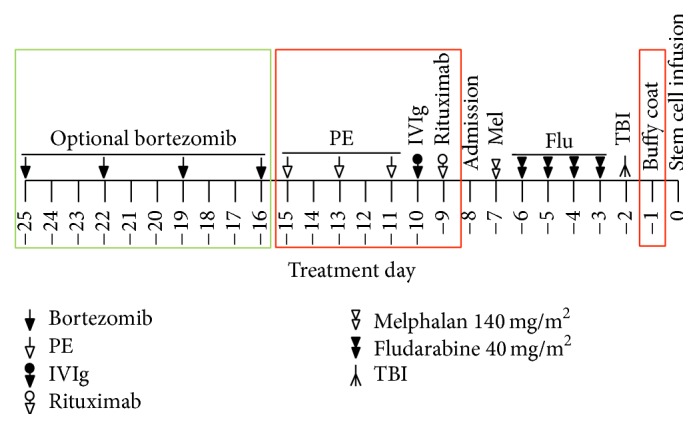
Desensitization approach for patients with DSAs undergoing haploidentical stem cell transplantation at MD Anderson Cancer Center.

Preformed antibodies present at the time of stem cell infusion are unaffected by standard transplantation conditioning regimens or T cell or B-cell immunosuppressive or modulatory strategies given in the peritransplantation period. To reduce the risk of GF, a number of studies have reported beneficial effects of a variety of interventions used to reduce total anti-HLA antibody load, predominantly by using a combined approach [66]. Reversal of DSAs mediated graft rejection and reduction in antibody load by using plasmapheresis, intravenous immunoglobulin (IVIg), cyclophosphamide, polyclonal anti-lymphocyte antibodies, monoclonal antibodies to CD20^+^ B lymphocytes (rituximab), and proteasome inhibitor against alloantibody producing plasma cells (bortezomib) have been described in solid organ transplant. However, their effectiveness is modest [67–71]. These treatment modalities also have been used to desensitize DSAs in haploSCT and mismatched AHSCT recipients with a variety of graft outcomes as summarized in Table 2. The first case was reported by Barge and colleagues in 1989; a patient with positive crossmatch test with donor lymphocytes was treated with plasmapheresis before haploSCT but did not result in a negative crossmatch before transplant and subsequently developed GF [41]. Maruta et al. confirmed that repeated high-volume plasmapheresis does not effectively eliminate preformed anti-HLA antibodies and applied adsorption of HLA-antibodies to irradiated donor lymphocytes before marrow transplantation for a successful engraftment [52]. We were the first to use a combined approach using plasmapheresis, IVIg, and rituximab with mixed results: out of the first 4 patients treated with this approach 2 achieved a significant reduction in antibody levels and engrafted the donor cells whereas the other 2 patients maintained high levels of DSAs and experienced primary GF [22]. Yoshihara et al. have tried 3 desensitization approaches for 5 patients who were to receive both bone marrow and peripheral blood stem cell grafts from haploidentical donors. Treatment regimen in this study was a combination of plasmapheresis, rituximab, antibody adsorption with platelets, and administration of the proteasome inhibitor, bortezomib. One of the 2 patients treated with plasmapheresis and rituximab received plasmapheresis on day −11 and the other received plasmapheresis on days −17, −15, and −13. Both were given a single dose of rituximab at 375 mg/mm^2^. DSA reduction was achieved in only 1 of 2 patients. However, both engrafted. Some of the most impressive reductions of DSAs were achieved by using 40 units of platelet transfusion from healthy donors selected to have the HLA antigens corresponding to the DSAs [48]. In a more recent study, in addition to 3 doses of alternating plasmapheresis every other day followed by 1 dose of IVIg and rituximab, we added an irradiated buffy coat infusion on day −1 prepared from 1 unit of blood on day −2 instead of using platelet transfusion to try to block remaining circulating antibodies after treatment as platelet has only class I HLA antigens on their surface (Figure 1) [46]. Moreover, in this study we have also found that what is more important appears to be the absence of C1q after treatment (conversion from C1q positivity to negativity) not merely the reduction of antibody levels. All 5 patients who remained C1q positive after treatment with plasmapheresis, IVIg, and rituximab with or without buffy coat prepared from donors experienced engraftment failure, whereas all 4 patients who became C1q negative after treatment/before transplant engrafted the donor cells. Although antibody level did not significantly change early on, all patients eventually clear the antibodies completely in the first few weeks after transplant [46]. These results suggested to us that a reduction to noncomplement binding level of DSAs should be the goal of treatment rather than clearing of the noncomplement binding DSAs, which appear to clear more slowly in the immediate posttransplant period and became undetectable in all patients within the first few weeks after transplant, similar to prior experience [72]. Although our experience is limited, this approach has been very successful as none of the patients treated as such experienced primary GF. A different approach was developed by the John Hopkins group from solid organ transplants, using a combination of repeated plasmapheresis, IVIg, and immunosuppressive medications. This group treated 15 mismatched AHSCT patients including 13 haploSCTs with alternate day of single volume plasmapheresis followed by 100 mg/kg of IVIg, tacrolimus (1 mg/day, i.v.), and mycophenolate mofetil (1 g twice daily) starting 1-2 weeks before the beginning of transplant conditioning, depending on patient's starting DSA levels. Reduction of DSA to the level that was thought safe for transplant was seen in 14 of 15 patients, all of these 14 patients engrafted with donor cells [56]. Even though, the majority of these studies have been anecdotal and included only a few patients but taken together have indicated that reduction of DSA to low levels can permit successful engraftment.

## 8. Conclusions

In the past 5 years much has been learned about the risks posed by DSAs in the development of primary GF in AHSCT with mismatched donors. These findings have impacted donor selection and helped the development of preventive treatments for allosensitized patients, who now can more safely undergo a transplant with a major HLA mismatched donor. Future studies will explore the pathogenesis of antibody-mediated rejection and develop effective therapies for allosensitized recipients.

## Figures and Tables

**Table 1:** DSAs and transplant outcomes.

Reference	Donor	Test	*N*	%Anti-HLA+	%DSA+	Graft outcome (DSA+/DSA−)	Comment
Ciurea et al. 2009 [22]	TCD HaploSCT	Luminex SA	24	NA	21	GF was 75% versus 5% (*P* = 0.008)	

Spellman et al. 2010 [23]	MMUD	FlowPRA, Luminex SA	115	37	8.7	24% of GF group had DSAs versus 1% of control group that had DSAs	

Ciurea et al. 2011 [3]	MUD, 1 Ag MMUD	Luminex SA	592	21	1.4	GF was 37.5% versus 2.7% (*P* = 0.0014)	

Yoshihara et al. 2012 [48]	HaploSCT	Luminex SA	79	20	14	GF was 27% versus 4% CI of neutrophil engraftment was 61.9% versus 94.4%, (*P* = 0.026)	(i) 5 patients were desensitized and 3/5 engrafted (ii) 67, 5, and 7 patients were antibody-negative, non-DSA-positive, and DSA-positive after desensitization

Ciurea et al. 2015 [46]	HaploSCT	Luminex SA	122	NA	18	GF was 32% versus 4% (*P* < 0.001)	

Chang et al. 2015 [49]	HaploSCT	NA	345	25.2	11.3	Primary graft rejection was 20% versus 0.3% (*P* = 0.002) Primary poor graft function was 27.3% versus 1.9% (*P* = 0.003)	

Takanashi et al. 2010 [50]	Single UCB	FlowPRA, Luminex SA	386	23.1	5	CI of neutrophil engraftment was 32% versus 83% (*P* < 0.0001)	Patients with DSA had significantly lower EFS and OS compared with no DSA

Brunstein et al. 2011 [51]	Double UCB	Luminex SA	126	41	24% had DSAs target to 1 UCB, 12% had DSA target to both UCB	GF was 17% versus 22%	

Cutler et al. 2011 [24]	Double UCB	Luminex SA	73	NA	24.6	GF was 18.2% and 57% in patients who had DSAs against 1 and 2 UCB, respectively, versus 5.5% in patients without DSAs (*P* = 0.01)	The rates of death or relapse within 100 days for the group of patients without DSAs, with DSAs against a single UCB unit, or DSAs against both UCB units were 23.6%, 36.4%, and 71.4%, respectively (*P* = 0.01)

Ruggeri et al. 2013 [39]	Single UCB, double UCB	Luminex SA	294	21	4.7	GF was 56% versus 23%	The presence of DSA was associated with lower survival (42% versus 29%; *P* = 0.07).

MMUD: mismatched unrelated donor; MUD: matched unrelated donor; GF: graft failure; DSA: donor specific antibody; TCD HaploSCT: T cell-depleted haploidentical stem cell transplant; UCB: umbilical cord blood; EFS: event-free survival; OS: overall survival; NA: not available.

**Table 2:** DSA desensitization in haploidentical and mismatched related AHSCT.

Reference	Donor type (*N*)	Anti-HLA abs test	Desensitization method	MFI after treatment	Graft outcome
Barge et al. 1989 [41]	Haplo (*N* = 1)	CDC	Plasmapheresis	NA	Graft failure

Maruta et al. 1991 [52]	Mismatched related (*N* = 1)	AHG-CDC	CyA, methylpred, Plasmapheresis, DLI	Negative XM	Engrafted

Braun et al. 2000 [53]	Haplo (*N* = 1)	FCXM	Staphylococcal protein A immunoadsorption	Negative XM	Engrafted

Ottinger et al. 2002 [20]	Mismatched related (*N* = 2)	DTT-CDC	Plasmapheresis, mismatched platelet transfusion	1 patient with negative XM, 1 patient with positive XM	Patient with negative XM after treatment engrafted, while patients with positive XM had GF

Pollack and Ririe 2004 [54]	Mismatched HLA-A68 related (*N* = 1)	FCXM	Platelet transfusion, plasmapheresis, IVIg	Negative XM	Engrafted

Narimatsu et al. 2005 [55]	Mismatched related (*N* = 1)	AHG-LCT	Rituximab, platelet transfusion	Negative AHG-LCT	Engrafted

Ciurea et al. 2009 [22]	Haplo (*N* = 4)	Luminex MFI >500	Rituximab, plasmapheresis	1 negative, 1 low titer, 2 high titers	Patients with DSAs negative and low titer after treatment engrafted; 2 patients with high titer had GF

Yoshihara et al. 2012 [48]	Haplo (*N* = 5)	Luminex MFI >500	Plasmapheresis + rituximab (*N* = 2), platelet transfusion (*N* = 2), bortezomib + dexa (*N* = 1)	1 patient had temporary DSA reduction and 1 patient had significant reduction after plasmapheresis; 2 patients had a significant reduction post platelet transfusion; 1 patient had moderate DSA reduction after bortezomib and dexa	All patients engrafted

Ciurea et al. 2015 [46]	Haplo (*N* = 12)	Luminex MFI >500	Plasmapheresis + rituximab + IVIg (*N* = 5), PE + rituximab + IVIg + donor buffy coat infusion (*N* = 7)	No significant change of MFI before transplant All patients cleared DSA after transplant	5 patients with C1q positive after treatment had GF while patients who became C1q negative engrafted

Leffell et al. 2015 [56]	Haplo (*N* = 13) MMUD (*N* = 2)	Luminex MFI >1000	Plasmapheresis + IVIg	Mean reduction of DSAs after treatment was 64.4%. 1 patient failed to reduce DSAs to the level that was thought to be safe for transplant	All 14/14 transplanted patients engrafted

MFI: mean fluorescence intensity; CDC: complement mediated cytotoxic; XM: crossmatch, FCXM: flow cytometric crossmatch, GF: graft failure; AHG-LCT: anti-human immunoglobulin lymphocytotoxicity test; NA: not available; MMUD: mismatched unrelated donor.
